# Editorial: Women in science - geriatric medicine 2021

**DOI:** 10.3389/fmed.2022.1008061

**Published:** 2022-08-17

**Authors:** Graziamaria Corbi, Valeria Conti, Amelia Filippelli

**Affiliations:** ^1^Department of Translational Medical Sciences, University of Naples Federico II, Naples, Italy; ^2^Department of Medicine, Surgery and Dentistry “Scuola Medica Salernitana”, University of Salerno, Baronissi, Italy

**Keywords:** gender and sarcopenia, mortality in elderly, Levodopa gender differences, carotenoid and mortality, women in science, survival and cholesterol in elderly, frailty and services utilization

In a world where the gender gap is often mentioned, where the role of women in society is often discussed, but in the same world where the strength of differences seems to be the only reality, the Research Topic in “*Women in science - geriatric medicine 2021*” tried to check the pulse of the Women researchers in all fields of the Geriatric Medicine. This topic aimed to assemble all the latest knowledge and the most recent ongoing research that sees Women as the principal responsible for the projects.

In particular, from the identification of specific features of cardiac amyloidosis, through the definition of factors associated with frailty in different settings and countries, the effect of Low and High-Density Lipoprotein Cholesterol or the use of beta-carotene on Mortality, or the medication Use with the incidence of Dementia After COVID-19 Hospitalization, to the definition of the differences in the LEVO-DOPA pharmacokinetics by gender, and the development of a new conceptual definition of severe self-neglect, the Research Topic “*Women in science - geriatric medicine 2021*” tries to explore some of the main aspects of Geriatrics, focusing on all the aspects of the elderly life.

Starting with the effects of the calcific aortic stenosis in the geriatric population, Myasoedova et al. analyze the characteristics of patients with this condition in association with cardiac amyloidosis, and the possible impact of cardiac amyloidosis on mortality of the patients with aortic stenosis, and the effect of different treatment strategies on outcomes of patients with aortic stenosis and concomitant cardiac amyloidosis. The authors suggest that several specific clinical, electrocardiographic, and echocardiographic features can be considered “red flags” of cardiac amyloidosis in patients with aortic stenosis (Myasoedova et al.).

Then, some articles focused on the factors that can conditionate the functional capacity of the elderly, with particular interest for the osteosarcopenia in Community-Dwelling Mexicans (López-Teros et al.), and Chinese older population (Chen et al.), but also by big data analysis of Older Adults Hospitalized in Long-Term Care in Portugal (Ramos et al.).

Moreover, Moreno et al. investigated if sarcopenia influences the healthy life expectancy (HLE) and unhealthy life expectancy (ULE) among older adults from Santiago, Chile. The authors demonstrated sex differences in disability trajectories among sarcopenic older people.

Indeed, gender differences were also found by Conti et al. that investigated the gender-related differences in Levodopa pharmacokinetics in patients with Parkinson's disease at their first-ever intake of Levodopa. Women showed higher levels of AUC and Cmax when compared with men, but also multiple linear regression analyses showed that the female sex was the only predictor of AUC and Cmax.

Yuan et al. examined the association between frailty and inpatient services utilization and the mediating role of multimorbidity in the association between frailty and inpatient services utilization among older adults in rural China. The authors found that frailty among Chinese rural older adults is associated with higher inpatient services utilization, and multimorbidity mediates this association.

Then, the importance of other factors in modifying the survival of the elderly was investigated. By using the Shanghai Aging Study, Wu et al. investigated the association between the low and high-density lipoprotein cholesterol levels and 10-Year Mortality in Community-Dwelling Older adults. An inverse association of LDL and a U-shape relationship of HDL-C with long-term all-cause mortality was found in a cohort of community-dwelling older Chinese adults.

Starting by the evidence of the beneficial effects of some diet foods on humans (1–3), by performing a metanalysis, Corbi et al. checked the association between beta-carotene supplementation and mortality. The authors found no evidence of an overall preventive effect of beta-carotene supplements on total, cancer, CVD, and cerebrovascular mortality risk in a meta-analysis of RCTs published over the past 25 years. Instead, beta-carotene supplementation increased the risk of lung cancer mortality but decreased the risk of HIV-related mortality.

Finally, two other articles included in this topic faced two different aspects. Pickens et al. purposed a new definition for severe self-neglect with the development of a conceptual framework by Modifying the CREST model for Self-Neglect. Freudenberg-Hua et al. determined the 1-year incidence rate of post-COVID dementia; assessed the association between pre-COVID psychotropic medication use and post-COVID incident dementia; and explored the association between different classes and types of psychotropic medications and post-COVID incident dementia. In this cohort study of older adults hospitalized with COVID-19 at a large health system in New York, exposure to pre-COVID psychotropic medications was associated with a greater 1-year incidence of post-COVID dementia.

Finally, the Research Topic “*Women in science - geriatric medicine 2021*” shows the great involvement of women in all fields of research, including biological, clinical, therapeutical, and preventive aspects.

## Author contributions

GC contributed to the conception. VC and AF contributed to the design of the Research Topic. All authors contributed to the management of submission, the revision process, and the control of the articles quality. All authors contributed to the article and approved the submitted version.

## Conflict of interest

The authors declare that the research was conducted in the absence of any commercial or financial relationships that could be construed as a potential conflict of interest.

## Publisher's note

All claims expressed in this article are solely those of the authors and do not necessarily represent those of their affiliated organizations, or those of the publisher, the editors and the reviewers. Any product that may be evaluated in this article, or claim that may be made by its manufacturer, is not guaranteed or endorsed by the publisher.

## References

[1] Davinelli S Corbi G Zarrelli A Arisi M Calzavara-Pinton P Grassi D. Short-term supplementation with flavanol-rich cocoa improves lipid profile, antioxidant status and positively influences the AA/EPA ratio in healthy subjects. J Nutr Biochem. (2018) 61:33–9. 10.1016/j.jnutbio.2018.07.011 30179727

[2] Davinelli S Corbi G Righetti S Sears B Olarte HH Grassi D. Cardioprotection by cocoa polyphenols and ω-3 fatty acids: a disease-prevention perspective on aging-associated cardiovascular risk. J Med Food. (2018) 21:1060–9. 10.1089/jmf.2018.0002 29723102 PMC6206547

[3] Corbi G Conti V Komici K Manzo V Filippelli A Palazzo M. Phenolic plant extracts induce SIRT1 activity and increase antioxidant levels in the rabbit's heart and liver. Oxid Med Cell Longev. (2018) 2018:2731289. 10.1155/2018/2731289 30116475 PMC6079382

